# A Comparative Study of Erector Spinae Plane Block and Thoracic Epidural Block on Respiratory, Analgesic, and Hemodynamic Outcomes in Patients With Traumatic Rib Fractures

**DOI:** 10.7759/cureus.84309

**Published:** 2025-05-17

**Authors:** Manoj Kumar, Rakesh Bahadur Singh, Atit Kumar, Alankrita Acharya

**Affiliations:** 1 Anesthesiology, Uttar Pradesh University of Medical Sciences, Etawah, IND; 2 Anesthesiology, Autonomous State Medical College, Auraiya, IND

**Keywords:** erector spinae plane block, mean opioid consumption, rib fracture, thoracic epidural analgesia, visual analog score

## Abstract

Background

Rib fractures are associated with significant morbidity and mortality. Thoracic epidural analgesia (TEA) provides excellent pain relief in the management of rib fractures. However, side effects, such as hypotension, and the technical challenges associated with its insertion can limit the use of this technique. Erector spinae plane block (ESPB) is a more superficial ultrasound-guided block, which is easier to perform and does not pose the same risk factors. The primary objective of this study was to compare the analgesic efficacy of continuous TEA and continuous ESPB in patients with traumatic rib fractures, measured by pain scores using the visual analog scale (VAS) and mean opioid consumption, expressed in intravenous morphine equivalents (IME). Secondary objectives included evaluating respiratory and hemodynamic parameters and assessing adverse effects.

Materials and methods

This was a retrospective cohort study including 100 patients aged 18 years or more with a diagnosis of unilateral multiple rib fractures, who received either continuous TEA or continuous thoracic ESPB as a part of their pain management. Patient data were collected from the medical records of individuals admitted to the emergency department. Groups were assigned later based on the analgesic technique received: Group T (TEA) and Group E (ESPB), with interventions performed according to the institutional protocol. Patients in Group T received TEA in the sitting or lateral position using an 18-gauge Tuohy needle, with a 20-gauge epidural catheter placed for continuous infusion. A primary bolus dose of 15 ml bupivacaine 0.125% was administered, followed by continuous infusion at a rate of 0.1 ml/kg/hour for 48 hours. Patients in Group E received ultrasound-guided ESPB using a low-frequency transducer, with a 20-gauge catheter inserted through an 18-gauge Tuohy needle. A bolus dose of 20 ml of bupivacaine 0.125% was given, followed by continuous infusion at a rate of 0.1 ml/kg/hour for 48 hours.

Pain scores using VAS, mean opioid consumption, inspiratory peak volumes as measured with an incentive spirometer, and hemodynamic variables (heart rate, mean arterial pressure, and oxygen saturation (SpO2)) were recorded at baseline (0 hours) and at 3, 6, 12, 24, 36, and 48 hours post-procedure.

Results

The mean VAS scores were comparable between patients who received thoracic epidural analgesia and those who received ESPB across all time points. Additionally, there was no significant difference between the two groups in terms of mean opioid consumption, mean incentive spirometry volumes, and hemodynamic parameters, including heart rate, mean arterial pressure, and oxygen saturation (SpO₂).

Conclusion

The study concluded that ESPB appears to be a promising alternative to TEA, offering a simpler and safer approach to analgesia in patients with traumatic rib fractures.

## Introduction

Rib fractures are seen in more than 50% of patients presenting with blunt chest trauma and are associated with significant morbidity and mortality [1]. Inadequate analgesia in these patients can subsequently lead to the need for mechanical ventilation. Opioid-based intravenous analgesia is widely used for relieving pain caused by traumatic rib fractures [2]. Other modalities of pain relief in traumatic rib fractures are thoracic epidural blocks, intercostal nerve blocks, paravertebral block, and new emerging myofascial plane block like erector spinae plane block (ESPB) [3].

Thoracic epidural analgesia (TEA) is a valuable option for managing pain in patients with rib fractures. However, its use may be restricted due to certain patient-related factors such as coagulopathy, spinal deformities, or unstable hemodynamics. Additionally, potential complications, including hypotension, epidural hematoma, infection, and post-dural puncture headache from accidental dural puncture, must be considered [4]. Many trauma patients have injuries that contraindicate epidural use or prevent proper positioning for insertion. These include spinal injuries restricting flexion, lower limb fractures in traction, chest trauma causing severe pain, and long bone fractures with massive blood loss leading to hemodynamic instability. Thoracic epidural catheter insertion is also comparatively difficult.

Erector spinae plane block, a recent technique that was first reported in 2016 [5], may be an alternative to thoracic epidural block for providing thoracic analgesia. The ESPB is a superficial, ultrasound-guided technique known for its speed, ease of administration, and reduced patient discomfort. It is typically performed using an in-plane ultrasound approach, targeting the space between the erector spinae muscle and the thoracic transverse processes. By delivering local anesthetic at this site, the block affects both the dorsal and ventral rami of the thoracic and abdominal spinal nerves, resulting in a multi-dermatomal sensory blockade that covers the anterior, posterior, and lateral regions of the thoracic and abdominal walls [5]. ESPB is a safer alternative to TEA, with fewer complications due to its superficial approach. While TEA carries a higher risk of serious infections like epidural abscess, often caused by Staphylococcus aureus, ESPB minimizes this risk. Additionally, ESPB has a lower risk of bleeding compared to TEA and is less likely to cause severe complications, with its primary concerns being local anesthetic systemic toxicity from inadvertent intravascular injection and pneumothorax [6]. Moreover, unlike TEA, ESPB does not penetrate the dura and therefore cannot cause post-dural puncture headache.

A literature review revealed very few studies comparing TEA and ultrasound-guided ESPB for analgesia in traumatic rib fractures [7,8], with no such study available at the start of our research. Therefore, we designed this study to evaluate and compare the analgesic efficacy of TEA and ESPB, hypothesizing that ESPB would offer pain relief comparable to TEA while also improving respiratory and hemodynamic outcomes and potentially offering advantages in terms of safety and ease of administration. The primary objective is to compare the analgesic efficacy of the ESPB and TEA in patients with traumatic rib fractures, measured by pain scores using the visual analog scale (VAS) and mean opioid consumption, expressed in intravenous morphine equivalents (IME). Secondary objectives include evaluating respiratory and hemodynamic parameters as well as assessing adverse effects.

## Materials and methods

Study design and patient selection

**Figure 1:**
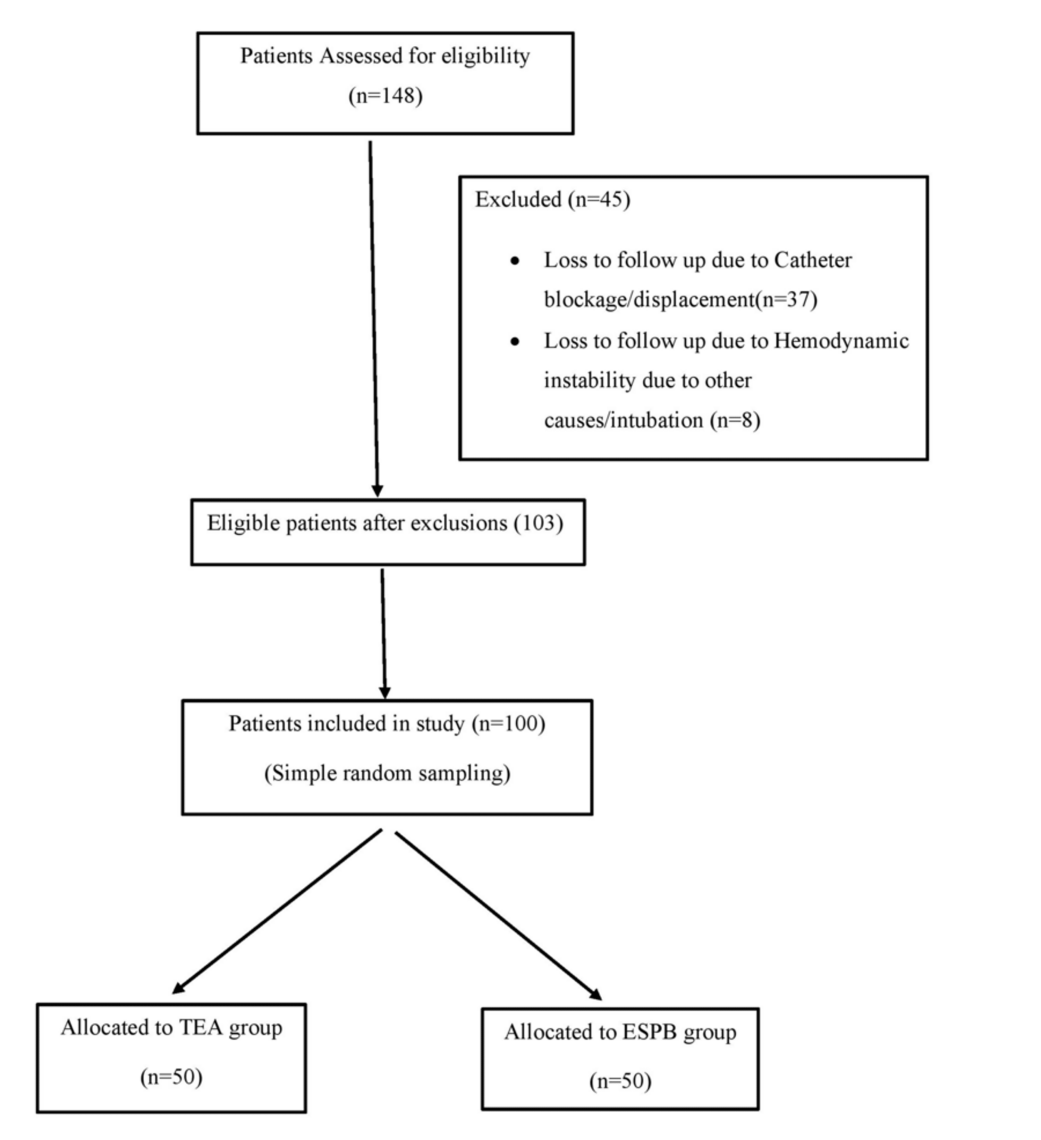
Flow diagram for the selection of study participants

After obtaining approval from the institutional ethics committee of Uttar Pradesh University of Medical Sciences, Saifai, Etawah (Ref. no. 1880/UPUMS/DEAN(M)/2020-21/E.C. NO:148/2020-21) and written informed consent from patients, we conducted a retrospective study in the department of anesthesiology between January 2020 and November 2021. The study included patients of either sex, aged over 18 years, with unilateral multiple rib fractures with or without chest injury, including pulmonary contusions, pneumothorax, and hemothorax. In this study, unilateral multiple rib fractures were defined as fractures involving two or more ribs on the same side, confirmed by radiological imaging (chest X-ray and/or CT scan). Patients with upper or lower limb injuries were included. However, patients with intra-abdominal, pelvic, or head injuries were excluded to ensure a homogenous study population and minimize confounding variables, as these injuries can significantly influence pain perception, opioid requirements, and respiratory parameters, potentially affecting the study outcomes. Patients experiencing a failure of the block or catheter dislocation during the study were also excluded. A total of 148 patients were screened, out of which 103 met the inclusion criteria. For ease of calculation, 100 patients were included in the final analysis (Figure 1).

Pain management protocol

At our institute, pain management for rib fractures follows a standardized protocol. Patients with rib fractures who are hemodynamically stable are admitted to the general ward. Initial pain management consists of intravenous (IV) diclofenac (75 mg) 8 hourly as the first-line analgesic. If pain persists (VAS > 4), tramadol 100 mg is administered every 8 hours. If pain remains uncontrolled (VAS > 4) despite this regimen, patients are transferred to the ICU within 24 hours for interventional pain therapy. Interventional pain management includes regional anaesthesia techniques, such as continuous TEA and continuous ESPB, which are administered by trained anesthesiologists with expertise in regional anaesthesia. If pain scores become manageable, injection tramadol is discontinued. For breakthrough pain, opioid analgesics are administered as needed.

Thoracic Epidural Analgesia (TEA)

In this group, patients received continuous TEA. The optimal site for epidural catheter insertion was determined with the patient in a sitting or lateral position, targeting the midpoint of the fractured ribs. The epidural space was identified using an 18 G Tuohy needle (Romsons Epi Kit®, GS-2026, Romsons Group Private Limited, Agra, India), followed by the insertion of a 20 G epidural catheter. An initial bolus of 15 ml of 0.125% bupivacaine was administered, followed by a continuous infusion at 0.1 ml/kg/hour for 48 hours.

Ultrasound-Guided Erector Spinae Plane Block (ESPB)

Patients in this group underwent ESPB using ultrasound (Sonosite M-Turbo, Fujifilm Medical Systems, Lexington, Massachusetts, US). The target vertebral level corresponded to the midpoint of the fractured ribs. Using a high-frequency (6-13 MHz) linear array transducer, the tip of the target vertebra was identified with the patient in a seated or lateral position. The transducer was placed in a parasagittal plane approximately 3 cm lateral to the midline, superficial to the erector spinae muscle. After local skin infiltration, an 18-G Tuohy needle used for epidural space identification was advanced in-plane with the ultrasound beam in a cranio-caudal direction until its tip was positioned between the erector spinae muscles and the transverse process. A 20-G catheter was then inserted through the needle. A bolus of 20 ml of 0.125% bupivacaine was injected, followed by a continuous infusion at 0.1 ml/kg/hour for 48 hours [6].

Analgesia Assessment

The effectiveness of analgesia was evaluated 20 minutes post-block using the loss of pinprick sensation. Failure was defined as the absence of sensory block in the corresponding dermatome, leading to exclusion from the study.

Adjunctive Analgesia

All patients received adjunctive analgesia during the 48-hour infusion period. Diclofenac (75 mg) every 8 hours was administered as the first-line agent while opioids (tramadol 100 mg, butorphanol 2 mg, fentanyl 100 mcg, or pentazocine 20 mg) were provided as rescue analgesia based on predefined pain management criteria (VAS > 4).

Study objectives

We defined the time point immediately before the administration of the regional block as baseline/0 hour, representing the pre-intervention pain assessment and serving as the reference for subsequent evaluations.

The primary objective was to assess pain scores using the VAS at 0, 3, 6, 12, 24, 36, and 48 hours post-block. Additionally, the mean opioid consumption, calculated as intravenous morphine equivalents (IME), was also a primary objective and was recorded at the same intervals.

To accurately assess opioid consumption in terms of IME, standardized conversion factors were used to compare different opioids by converting their doses into an equivalent amount of intravenous morphine. The following conversion factors were applied: Tramadol: 100 mg = 10 mg morphine IV; Butorphanol: 2 mg = 6.67 mg morphine IV; Fentanyl: 100 mcg = 10 mg morphine IV; Pentazocine: 20 mg = 6.67 mg morphine IV.

These conversion factors were based on established equianalgesic dose recommendations to ensure accurate opioid consumption assessment [9].

Incentive spirometry using a volume-oriented spirometer was employed to assess inspiratory peak volume (IPV), defined as the maximum volume of air (in millilitres) a patient can inhale following a full exhalation. This measurement served as an indicator of lung expansion and ventilatory function. IPV was recorded at baseline (0 hours, pre-intervention) and 3, 6, 12, 24, 36, and 48 hours post-block. Mean arterial pressure (MAP), heart rate (HR), and SpO₂ were recorded at baseline and at each designated time point. Any insertion site infections or cardiovascular issues (hypotension, bradycardia) were documented. Hypotension was defined as a decrease in mean arterial pressure (MAP) of more than 20% from baseline. Bradycardia was defined as a heart rate < 50 beats per minute (bpm) or a decrease of more than 20% from baseline.

Individuals measuring pain scores, respiratory function, or opioid consumption were blinded to the intervention received by patients to reduce bias. The statistician analyzing the data was also blinded to the group assignments to ensure unbiased analysis.

Statistical analysis

Statistical analysis was performed using SPSS version 23.0 (IBM Corp, Armonk, NY, USA). Descriptive statistics were applied to summarize patient demographics and baseline characteristics. Continuous variables were presented as mean ± standard deviation (SD). Categorical variables were reported as frequencies and percentages and analyzed using the chi-square test or Fisher’s exact test, as appropriate. The VAS score, opioid consumption, inspiratory peak volume, heart rate, mean arterial pressure, and SpO2 levels between the two groups were compared using an independent t-test. A p-value of <0.05 was considered statistically significant for all statistics.

## Results

A total of 100 patients were evaluated in this study. They were divided into two groups. Group T received TEA, and Group E received ESPB. The mean age of participants in Group T was 46 ± 11.38 years, while that in Group E was 42.52 ± 12.89 years. The mean BMI of participant group T was 24.79 ± 3.32 kg/m^2^, while that of group E was 24.83 ± 3.14 kg/m^2^. There was no significant difference in age, body mass index (BMI), and gender between the groups on statistical analysis (p>0.05). Chest injuries were the most common, observed in 78% vs. 86% (p = 0.475). Lower limb injuries were reported in 42% vs. 46% (p = 0.671) while upper limb injuries were noted in 32% vs. 38% (p = 0.523) (Table 1).

**Table 1:** Demographic data of study groups n = number of patients; Group T = Thoracic epidural block, Group E = USG-guided erector spinae plane block SD = Standard deviation; BMI = Body mass index; USG = Ultrasonography

Variable	Group T (n=50)	Group E (n=50)	p-value
Age (years)	46 ± 11.38	42.52 ± 12.89	0.078
BMI (kg/m²)	24.79 ± 3.32	24.83 ± 3.14	0.475
Gender (Male/Female)	41/9	44/6	0.200
Other Injuries			
- Upper Limb Injury	16 (32%)	19 (38%)	0.523
- Lower Limb Injury	21 (42%)	23 (46%)	0.671
- Chest Injury	39 (78%)	43 (86%)	0.475

The mean VAS score at 0 hour in group T was 6.06 ± 0.77, and in group E, it was 6.00 ± 0.93. The mean VAS score decreased to 2.14 ± 1.2 in Group T and to 2.26 ± 1.32 in Group E at 24 hours. At the end of 48 hours, the mean VAS score of both groups further decreased to 1.66 ± 0.92 and 1.70 ± 1.04, respectively. There is no statistically significant difference between the two groups (p>0.01) (adjusted α) (Table 2).

**Table 2:** Comparison of pain between groups T and E according to visual analog scale scores Group T = Thoracic epidural block, Group E = USG-guided erector spinae plane block SD = Standard deviation; USG = Ultrasonography

Visual Analog Scale Score	Group T	Group E	P-value
	Mean ± SD	Mean ± SD	
0 hour	6.06 ± 0.77	6.00 ± 0.93	0.362
3 hours	2.28 ± 1.07	2.12 ± 1.14	0.235
6 hours	2.52 ± 1.18	2.72 ± 1.47	0.228
12 hours	2.58 ± 1.3	2.56 ± 1.47	0.471
24 hours	2.14 ± 1.2	2.26 ± 1.32	0.318
36 hours	2.08 ± 1.1	2.22 ± 1.25	0.277
48 hours	1.66 ± 0.92	1.70 ± 1.04	0.419

The mean opioid consumption initially at 0 hour was 14.80 ± 1.41 in Group T and 15.00 ± 0 in Group E. The mean consumption of opioids decreased to 3.80 ± 5.21 at 24 hours and to 2.10 ± 4.53 at 48 hours in Group T, while in Group E, it decreased to 4.90 ± 6.51 and 2.90 ± 5.81 at 24 and 48 hours, respectively. There was no statistical difference between the groups (p>0.01) (adjusted α). Total opioid consumption, expressed in intravenous morphine equivalents (IME), was 35.50 ± 11.88 in the T group and 38.00 ± 13.45 in the E group, with no statistically significant difference between the two groups (p = 0.825) (Table 3).

**Table 3:** Comparison of mean opioid consumption in intravenous morphine equivalents (IME) between groups T and E Group T = Thoracic epidural block, Group E = USG-guided erector spinae plane block SD = Standard deviation; IME = Intravenous morphine equivalents; USG = Ultrasonography

Mean Opioid Consumption (IME)	Group T (Mean ± SD)	Group E (Mean ± SD)	p-value
0 hour	14.80 ± 1.41	15.00 ± 0	0.160
3 hours	2.20 ± 4.06	1.80 ± 3.61	0.302
6 hours	3.90 ± 4.44	4.00 ± 4.52	0.456
12 hours	4.80 ± 5.25	5.80 ± 5.75	0.183
24 hours	3.80 ± 5.21	4.90 ± 6.51	0.176
36 hours	3.70 ± 5.03	4.60 ± 6.30	0.216
48 hours	2.10 ± 4.53	2.90 ± 5.81	0.222
Total Opioid Consumption (IME)	35.50 ± 11.88	38.00 ± 13.45	0.825

The mean inspiratory peak volume (ml) at 0 hour was 760.00 ± 312.89 in Group T and 788.00 ± 330.72 in Group E. The mean inspiratory peak volume increased to 1206.00 ± 315.73 at 24 hours and to 1311.00 ± 351.78 at 48 hours in Group T, while in Group E, it increased to 1350.00 ± 342.30 at 24 hours and to 1488.00 ± 373.28 in Group E at 48 hours. There was no statistical difference between the groups (Table 4).

**Table 4:** Comparison of inspiratory peak volume (ML) between groups T and E Group T = Thoracic epidural block, Group E = USG-guided erector spinae plane block SD = Standard deviation; USG = Ultrasonography

Inspiratory Peak Volume (ml)	Group T	Group E	p-value
	Mean ± SD	Mean ± SD	
0 hour	760.00 ± 312.89	788.00 ± 330.72	0.408
3 hours	1020.00 ± 310.37	998.00 ± 336.43	0.403
6 hours	1125.00 ± 321.31	1148.00 ± 339.87	0.393
12 hours	1170.00 ± 318.84	1234.00 ± 339.97	0.404
24 hours	1206.00 ± 315.73	1350.00 ± 342.30	0.416
36 hours	1270.00 ± 325.14	1412.00 ± 354.42	0.459
48 hours	1311.00 ± 351.78	1448.00 ± 373.28	0.408

The mean heart rate (beats per min) at 0 hour was 108.60 ± 15.48 in Group T and 108.42±15.68 in Group E. The mean heart rate decreased to 82.46 ± 15.89 at 24 hours and 72.18 ± 14.84 at 48 hours in Group T, while in Group E, it decreased to 93.92 ± 15.35 at 24 hours and to 88.90 ± 15.64 in Group E at 48 hours. No statistical difference was observed between the study groups (Table 5).

**Table 5:** Comparison of heart rates (beats per min) between groups T and E Group T = Thoracic epidural block, Group E = USG-guided erector spinae plane block SD = Standard deviation; USG = Ultrasonography

Heart Rate (beats per min)	Group T	Group E	p-value
	Mean ± SD	Mean ± SD	
0 hour	108.60 ± 15.48	108.42 ± 15.68	0.477
3 hours	97.38 ± 14.56	96.96 ± 14.6	0.443
6 hours	90.14 ± 14.58	94.98 ± 14.29	0.486
12 hours	88.34 ± 13.19	93.70 ± 14.03	0.491
24 hours	82.46 ± 15.89	93.92 ± 15.36	0.452
36 hours	78.26 ± 15.19	91.52 ± 15.35	0.471
48 hours	72.18 ± 14.84	88.90 ± 15.64	0.357

The mean arterial pressure (mmHg) at 0 hour in Group E was 107.44 ± 9.07 and 107.76 ± 9.78 in Group T. The mean arterial pressure decreased to 96.12 ± 8.32 at 24 hours and 94.80 ± 7.88 at 48 hours in Group T, while it decreased to 96.14 ± 8.61 at 24 hours and 94.58 ± 8.17 at 48 hours in Group E. No statistical difference was observed between the two groups (p > 0.05) (Table 6).

**Table 6:** Comparison of mean arterial pressure (mmHg) between groups T and E Group T = Thoracic epidural block, Group E = USG-guided erector spinae plane block SD = Standard deviation; USG = Ultrasonography

Mean Arterial Pressure (mmHg)	Group T	Group E	p-value
	Mean ± SD	Mean ± SD	
0 hour	107.44 ± 9.07	107.76 ± 9.78	0.433
3 hours	97.66 ± 9.55	97.06 ± 9.24	0.375
6 hours	97.10 ± 7.76	96.66 ± 7.94	0.390
12 hours	94.76 ± 8.36	94.32 ± 8.01	0.394
24 hours	96.12 ± 8.32	96.14 ± 8.61	0.495
36 hours	95.32 ± 7.83	95.92 ± 8.33	0.356
48 hours	94.80 ± 7.88	94.58 ± 8.17	0.446

The mean SpO2 (%) at 0 hour was 93.84 ± 2.41 in Group T and 93.72 ± 2.52 in Group E. The mean SpO2 (%) increased to 95.20 ± 2.41 at 24 hours and 95.86 ± 2.73 at 48 hours in Group T, while in Group E, it increased to 94.96 ± 2.65 at 24 hours and 95.52 ± 2.84 at 48 hours. No statistical difference was observed between the groups (Table 7).

**Table 7:** Comparison of SpO2 (%) between groups T and E Group T = Thoracic epidural block, Group E = USG-guided erector spinae plane block SD = Standard deviation; SpO2 = Saturation of peripheral oxygen; USG = Ultrasonography

SpO_2_ (%)	Group T	Group E	p-value
	Mean ± SD	Mean ± SD	
0 hour	93.84 ± 2.41	93.72 ± 2.52	0.404
3 hours	94.32 ± 2.38	94.24 ± 2.45	0.434
6 hours	94.70 ± 2.48	94.60 ± 2.60	0.422
12 hours	95.04 ± 2.32	94.90 ± 2.55	0.387
24 hours	95.20 ± 2.41	94.96 ± 2.65	0.318
36 hours	95.78 ± 2.43	95.40 ± 2.73	0.232
48 hours	95.86 ± 2.73	95.52 ± 2.84	0.272

## Discussion

Pain due to a rib fracture can interfere with the respiratory physiology of a patient. It can result in reduced chest movement, atelectasis, pneumonic consolidation, etc. [10]. Hence, after stabilizing patients with traumatic rib fractures, our main focus should always be on relieving patients of their pain. There are various analgesic modalities available for patients with traumatic rib fractures, including oral and intravenous drugs, thoracic epidural blocks, paravertebral blocks, and ultrasonography (USG)-guided fascial plane blocks. Thoracic epidural analgesia has been routinely used in patients with traumatic rib fractures; however, it has its own drawbacks, as it carries a higher risk of hypotension due to sympathetic blockade, potential motor weakness, more technical challenges in placement, and a greater likelihood of complications such as epidural hematoma, dural puncture, and catheter-related infections. However, USG fascial plane blocks have recently emerged as excellent alternatives for analgesia in patients with traumatic rib fractures.

This study was conducted to compare the TEA with USG-guided ESPB for analgesia in patients with traumatic rib fractures. VAS scores for pain and mean opioid consumption (calculated as IME) were observed at 0, 3, 6, 12, 24, 36, and 48 hours after the blocks were administered. The incentive respiratory volumes, the hemodynamic variables (mean arterial pressure, heart rate, SpO2), and complications were also observed. In our study, the VAS score decreased in both groups over 48 hours. The results were comparable between the study groups. The comparable VAS scores between TEA and ESPB groups may be due to effective analgesia in both techniques, standardized adjunctive analgesia, similar injury severity, and the widespread local anesthetic spread in ESPB. Nagaraja PS et al. conducted a study in 50 patients to compare continuous TEA with bilateral ESPB for perioperative pain management in cardiac surgery [11]. Both groups had a comparable VAS score at 0, 3, 6, and 12 hours (p> 0.05), but there was a statistically significant (p≤0.05) difference in VAS scores, which was lower in the ESPB at 24, 36, and 48 hours. However, in our study, the VAS score was found to be comparable between both groups at all time intervals.

In concordance with our study, other related studies proved that ESPB is effective in pain management in patients with rib fractures. Abdelraheem Elawamy et al. conducted a clinical trial to compare ESPB with thoracic paravertebral block for pain management in 60 patients with unilateral multiple fractured ribs and found that VAS scores at rest and on coughing were comparable between the groups at all measuring points except at 0.5 hours following the block performance, and concluded that ultrasound-guided thoracic ESPB was as effective as thoracic paravertebral block for pain alleviation in patients with unilateral multiple fractured ribs with a comparable duration of analgesic effect, reduction of opioid consumption, and stable hemodynamic profile [12]. Another prospective descriptive study by Rashmi Syal et al. on continuous erector spinae plane block for analgesia and better pulmonary functions in 10 patients with multiple rib fractures, found that a continuous ESP block provides a significant reduction in pain scores in patients with multiple rib fractures [13]. Adhikary SD et al. conducted a study on the effect of erector spinae plane block on respiratory and analgesic outcomes in multiple rib fractures in 79 patients and found a significant reduction in maximum numerical pain rating scale (NRS) scores following erector spinae plane blockade [14].

In our study, mean opioid consumption was significantly decreased in both groups, and the results were comparable between group T and group E. This was similar to a study conducted by Nagaraja PS et al., who found that total intraoperative fentanyl consumption was comparable between the two groups in perioperative pain management in cardiac surgeries [11]. However, none of the patients received rescue analgesia during the VAS assessment in his study. In our study, rescue analgesia was administered, which can be attributed to the fact that patients in our study had associated traumatic injuries. Another study was conducted by Adhikary SD et al., who observed reductions in opioid consumption after ESPB in patients with traumatic rib fractures and concluded that erector spinae plane blocks were associated with improved analgesic outcomes following rib fracture [14]. This is consistent with the results of our study.

The mean incentive spirometry peak volume improved in both groups, and results were comparable between the study groups. The comparable improvement in mean incentive spirometry peak volume between the study groups may be attributed to effective pain relief provided by both analgesic techniques, facilitating better respiratory effort and lung expansion. Our observations were consistent with the study conducted by Adhikary SD et al., who found that Incentive spirometry volumes improved from 784 (694) to 1375 (667) ml during the first 24 hours following erector spinae plane blockade [14]. Singh et al. conducted a randomized study on 50 patients to compare continuous thoracic epidural block and continuous thoracic paravertebral block in the management of thoracic trauma [15]. There was a significant improvement in pulmonary function tests in both groups post-procedure, which is similar to our results.

SpO2 (%) improved nearly equally in both groups, indicating that continuous TEA and ESPB offered comparable levels of pain relief and, consequently, similar respiratory support. This suggests that both techniques were almost equally effective in enhancing respiratory function in patients with traumatic rib fractures. In support of our study, Syal et al., in their study on continuous ESPB for analgesia and better pulmonary functions in 10 patients with multiple rib fractures, concluded that respiratory rate and SpO2 were significantly better after the block placement [13].

In the present study, a decrease in mean arterial pressure (MAP) was observed in both groups following the intervention. This indicates a reduction in blood pressure following the intervention. However, despite this decrease, none of the participants met the criteria for hypotension. This can be attributed to the exclusion of patients with hemodynamic instability or severe injuries, which are significant risk factors for profound hypotensive episodes. Additionally, no instances of bradycardia or catheter-related infections were observed.

Limitations

In this study, the impact of associated traumatic injuries on pain scores and opioid consumption could not be specifically evaluated, which may have influenced the findings. Additionally, oral analgesia was administered based on individual pain assessments and clinical discretion, leading to variability in opioid use. This variability is recognized as a potential confounding factor. However, since the primary focus was on comparing the efficacy of regional analgesic techniques (TEA vs. ESPB), opioid consumption was standardized using IME to facilitate an objective comparison between the groups. Moreover, since the study was carried out in a controlled hospital environment, its findings may not be directly applicable to other healthcare settings, particularly those with limited resources. Conducting multicentre trials with a larger sample size could enhance the generalizability and external validity of the results.

## Conclusions

From the present study, we concluded that the USG-guided ESPB, when administered, is as efficacious as TEA for analgesia in patients with traumatic rib fractures. ESPB holds promise as a simpler alternative technique in patients with traumatic rib fractures, where thoracic epidural catheters are technically difficult to insert. ESPB is a safer technique, as it also avoids potential complications that are associated with a thoracic epidural block, such as a post-dural puncture headache, epidural abscess, and epidural hematoma.
